# Practical Diagnosis

**Published:** 1903-02

**Authors:** 


Practical Diagnosis.—The use of symptoms in the diagnosis of disease. By Hobart Amory Hare, M. D., B. Sc., Professor of Therapeutics and Materia Medica in the Jefferson Medical College of Philadelphia. Fourth edition, enlarged and thoroughly revised. In one octavo of 727 pages, with 236 engravings and 24 full page colored plates. Cloth, $5.00, net. Leather, $6.00, net. Half Morocco, $7.00, net. Lea Brothers & Co., Publishers, Philadelphia and New York.

It is with the greatest interest we have just reviewed this edition of Hare's Practical Diagnosis and take pleasure in saying that each edition has added luster, combined with the most practical and invaluable information, until now, through its own merits it has acquired an indispensable place in every physician's and student's library. No library is complete without it. The accuracy with which the author deals with symptomatology, aiding the physician to arrive at a rational diagnosis, suggesting the possible cause and location of pathologic lesions in brain and spinal diseases desires special mention. Coma, unconsciousness, etc., are conditions frequently met with in such cases; the physician is often at a loss to find the cause. Under such circumstances, to hold a consultation with Dr. Hare's Practical Diagnosis gives new and better light and offers valuable suggestions. The various conditions physiologic and pathologic which may be suggested by nausea and vomiting or by headache, are recorded with the author's characteristic impressive style, showing concisely what each symptom may indicate and how often these apparently simple fore-runners are the bearer of the most valuable information, the prelude of disastrous consequences.

It is not surprising that this work has come to its fifth edition in six years, for in addition to its general practical value it is arranged with a view of maximum facility of reference, and the frequent revisions keep it up to the most recent advances on the subject with which it deals. T. P. L.

